# Flawed risk assessment of antifouling paints leads to exceedance of guideline values in Baltic Sea marinas

**DOI:** 10.1007/s11356-020-08973-0

**Published:** 2020-05-11

**Authors:** Maria Lagerström, João Ferreira, Erik Ytreberg, Ann-Kristin Eriksson-Wiklund

**Affiliations:** 1 Department of Environmental Science and Analytical Chemistry (ACES), Stockholm University, SE-106 91 Stockholm, Sweden; 2 Department of Mechanics and Maritime Sciences, Chalmers University of Technology, SE-412 96 Gothenburg, Sweden

**Keywords:** Antifouling paint, Copper, Zinc, Risk assessment, Baltic Sea

## Abstract

The seasonal variations of dissolved and bioavailable copper (Cu) and zinc (Zn) were studied in two recreational marinas in Sweden and Finland. The time series from the two marinas were characterized by rising concentrations during the spring boat launching, elevated concentrations all through the peak boating season, and decreasing concentrations in autumn when boats were retrieved for winter storage. This pattern shows a clear link between Cu and Zn concentrations and boating activity, with antifouling paints as the principal source. The leaching from antifouling paints was also found to significantly alter the speciation of dissolved Cu and Zn in marina waters, with an increase of the proportion of metals that may be considered bioavailable. This change in speciation, which occurred without any change in dissolved organic carbon (DOC), further increases the environmental risk posed by antifouling paints. In the Swedish marina, dissolved Cu and Zn exceed both Environmental Quality Standards (EQS) and Predicted No Effect Concentrations (PNEC), indicating that the current Swedish risk assessment (RA) of antifouling paints is failing to adequately protect the marine environment. An evaluation of the RA performance showed the underlying cause to be an underestimation of the predicted environmental concentration (PEC) by factors of 2 and 5 for Cu and Zn, respectively. For both metals, the use of inaccurate release rates for the PEC derivation was found to be either mainly (Cu) or partly (Zn) responsible for the underestimation. For Zn, the largest source of error seems to be the use of an inappropriate partitioning coefficient (*K*_D_) in the model. To ensure that the use of antifouling coatings does not adversely impact the sensitive Baltic Sea, it is thus recommended that the *K*_D_ value for Zn is revised and that representative release rates are used in the RA procedure.

**Electronic supplementary material:**

The online version of this article (10.1007/s11356-020-08973-0) contains supplementary material, which is available to authorized users.

## Introduction

The Baltic Sea, classified as a Particularly Sensitive Sea Area in 2001 by the International Maritime Organization (IMO), has specific ecological characteristics resulting in a limited but unique species diversity (Kachel 2008). This semi-enclosed sea is subject to multiple stressors such as eutrophication, overfishing, ocean acidification, and climate change, making its management both an intricate and vital issue (Jutterström et al. 2014; Elmgren et al. 2015). Contamination of hazardous substances is another well-known stressor, to which the leaching of biocides from antifouling paints is contributing. Antifouling paints are biocidal products used to deter fouling by aquatic organisms on underwater structures, e.g., submerged ship and boat hulls, through the leaching of toxins into the water phase (WHOI 1952). The potential of biocidal antifouling compounds to cause adverse effects on non-target organisms has been widely reported (Konstantinou and Albanis 2004; Thomas and Brooks 2010; Dafforn et al. 2011), and biocide-containing antifouling paints are currently regulated and require approval. In the European Union (EU), there are at present 14 active substances approved for use in antifouling products under the Biocidal Products Regulation (BPR) (European Parliament and Council 2012), and both the choice and leaching rate of the biocide(s) will determine a paint’s efficacy in preventing fouling.

Copper compounds, mainly cuprous oxide, have long been the primary biocides in antifouling paints and are still the most widely used today (WHOI 1952; Howell and Behrends 2010). Zinc oxide is a common seawater-soluble filler used to help control the polishing rate, especially in yacht paints (Yebra et al. 2006; Yebra and Weinell 2009; Lindgren et al. 2018). In marinas, it is therefore mainly these two metals, i.e., Cu and Zn, which are released from the paints and consequently contaminate both water and sediments (An and Kampbell 2003; Kylin and Haglund 2010; Briant et al. 2013; Boyle et al. 2016; Pourabadehei and Mulligan 2016). Changes in water quality through metal contamination have been shown to affect biological systems, leading to changes in the abundance and composition of sessile and mobile organisms (Perrett et al. 2006; Dafforn et al. 2008) and increased metal content in organism tissues (Johnston et al. 2011). The toxicities of Cu and Zn to marine species are controlled by the metals’ chemical speciation, determining their potential for biological uptake. The metals are released from antifouling paints in the form of bioavailable Cu^2+^ and Zn^2+^ (Yebra et al. 2006; Thomas and Brooks 2010; Howell and Behrends 2010). The bioavailability in the water phase is however reduced through complexation with inorganic and, most importantly, organic ligands (Brooks and Waldock 2009; Vraspir and Butler 2009). The complexation of Cu by organic matter and the effect of dissolved organic carbon (DOC) on Cu toxicity have been well studied, in particular (Arnold 2005).

Under the BPR, antifouling paints must pass an environmental risk assessment (RA) before their placement on the market. In the EU, this typically involves the modeling of predicted environmental concentrations (PECs) in marina waters of the active substances (e.g., Cu) and substances of concern (e.g., Zn) in the Marine Antifoulant Model to Predict Environmental Concentrations (MAMPEC). These are then evaluated against defined Predicted No Effect Concentration (PNEC) values. Only products which are not predicted to cause exceedance of the PNEC in the model marina, i.e., PEC/PNEC < 1, will be authorized. According to another regulation, the EU Water Framework Directive (WFD), Member States (MS) should strive for the attainment of “Good Status” in all water bodies (European Parliament and Council 2000). The status of a water body is defined by ecological and chemical parameters, with overall “Good Status” achieved when both good ecological and good chemical status are attained. For elements and compounds on the list of so-called Priority Substances, national Environmental Quality Standard (EQS) values to be met as part of the chemical status have to be established by each MS. MS may also identify river basin–specific pollutants (e.g., Cu and Zn) to be evaluated as a part of the ecological status of a water body. It is thus imperative that the RA performed with accordance to the BPR prevents the authorization of products which can jeopardize the attainment of “Good Status” under the WFD.

In this study, the impact of antifouling paints on the concentration and speciation of dissolved Cu and Zn in two Baltic Sea marinas was studied. Seasonal variations were measured through discrete water sampling and deployment of diffusive gradients in thin films (DGTs). DGTs are passive sampling devices which accumulate dissolved metals using a chelating gel, providing the average in situ concentration of metals in the water during the time of deployment (Davison and Zhang 1994). The DGT concentrations obtained reflect the speciation of metals as the devices only bind the dissolved species that are labile and considered available to biota (Zhang and Davison 2000; Twiss and Moffett 2002; Dunn et al. 2003; Munksgaard and Parry 2003; Forsberg et al. 2006; Zhang and Davison 2015). The objective of this study was to firstly investigate the seasonal variations of Cu and Zn (dissolved and bioavailable) in marinas and assess the status of the water quality. Ni was also measured for comparison, as it is a metal that does not originate from boating-related activities. Secondly, the ability of MAMPEC to predict environmental concentrations was assessed through the comparison of modeled PECs for some authorized Baltic Sea paints to the measured concentrations in one of the marinas. Potential reasons for the observed discrepancies between the two are investigated and improvements to the RA procedure are suggested.

## Materials and methods

### Study sites

**Fig. 1:**
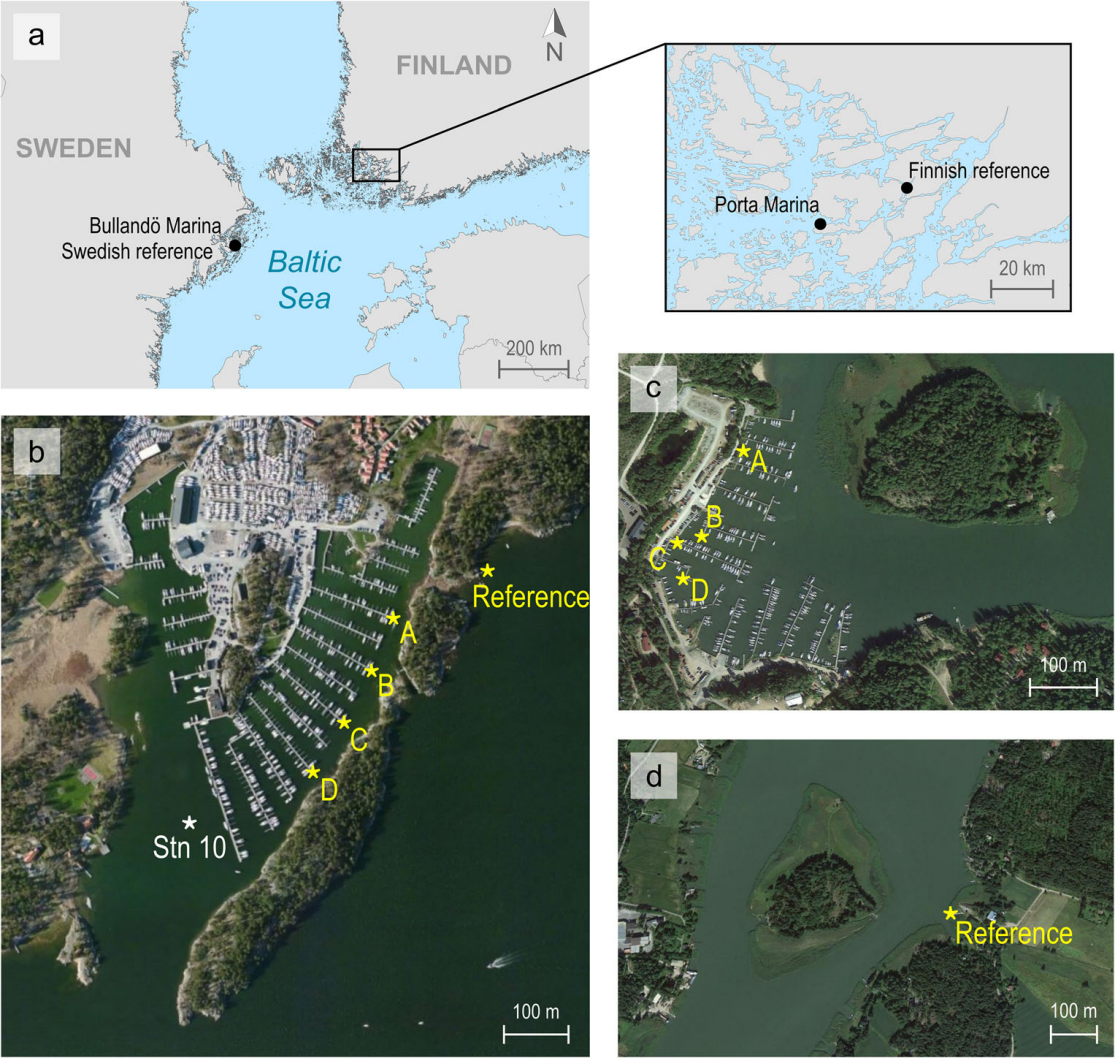
Map of the sampling locations (a) and aerial photos showing Bullandö Marina and the Swedish reference site ((b), image from Bing Maps, May 2012), Porta Marina ((c), Google Earth, Aug 2015), and the Finnish reference site ((d), Google Earth, Aug 2015). DGTs were deployed and grab water samples collected at four locations within each marina (A, B, C, and D). Also shown in aerial photo (b) is the location of station 10, sampled in previous studies in both 1993 and 2004

The study was performed in two recreational marinas in the Baltic Sea: Bullandö Marina (Stockholm archipelago, Sweden; 59.298° N, 18.653° E) and Porta Marina (Turku archipelago, Finland; 60.263° N, 22.121° E). Bullandö Marina is one of the largest marinas in Sweden with a berthing capacity of 1400 boats. Porta Marina is a medium-sized marina with approximately 330 berths but is located right next to another marina of similar capacity. The bay therefore hosts around 600 boats altogether. Both marinas are located in brackish waters with measured salinities of 5.1 and 5.8 PSU for Bullandö Marina and Porta Marina, respectively. One reference site close to each marina was also studied. Their locations were chosen so that the sites would be positioned as close as possible to the marinas to ensure similar water parameters, without being impacted by leisure boat activity or any other potential sources of metal contaminants. Figure 1 shows the location of all the study sites. At Bullandö Marina, all sampling locations were in the Eastern bay of the marina (Fig. 1b), where roughly 1200 boats are moored. The Swedish reference site was located adjacent to Bullandö Marina, only 900 m away from the marina inlet (Fig. 1b), whereas the Finnish reference site was located approximately 30 km away from Porta Marina (Fig. 1d). The time periods of boat launching and uptake for winter storage at the beginning and end of the boating season at the marinas were recorded through photos and notes about marina occupancy at each sampling event. Information was also gathered from the marina websites and/or through contact with the owners. At Bullandö Marina, the number of boats was also counted, along one of the jetties, at each sampling event in order to estimate occupancy.

### Passive samplers and discrete water sampling

The passive DGT samplers were acquired from DGT Research Ltd. (C-LSNM Loaded DGT device for metals (A) in solution, Lancaster, UK). Deployments of the devices were carried out at 4 locations (A, B, C, and D) within each marina and at 1 location at each reference site (Fig. 1b–d). In order to yield representative concentrations for the marina water as a whole, the sampling locations were selected so that the DGTs would be in the midst of the harbor, but not in direct vicinity of boats (nearest boat hull at least 2 m away). The DGTs were deployed at 1-m depth for intervals of 72 h at each deployment occasion. The exposure time of 72 h was chosen as it was estimated to be long enough to obtain concentrations above the detection limit while minimizing the risk of fouling on the samplers which can act to reduce the amount of analyte trapped in the binding gel (Davison 2016). Temperature loggers (HOBO Pendant® Temperature Logger, UA-002-08) were placed at all study sites to monitor the water temperature at 1-m depth. DGTs were deployed once a month during March–November 2016, with some additional deployments in April and May during the launching of the boats. Collection of grab water samples for DOC and dissolved metals (0.45 μm filtered) concurred with the DGT deployments. The sampling of dissolved metals was carried out according to trace metal clean procedures. At the Swedish sites, grab samples were collected at all DGT deployment occasions but only at three occasions at the Finnish sites (in March, July, and October). For both marinas, DOC samples were only collected at location “A” in the harbor.

### Chemical analyses

Concentrations of dissolved Cu, Zn, and Ni in the grab water samples were analyzed by an inductively coupled plasma-mass spectrometer (ICP-MS, NexION® 350D, PerkinElmer). The accuracy was verified through analysis of the estuarine reference water BCR-505 whose analyzed concentrations (average ± 1 standard deviation, *n* = 7) were all within the certified range: 1.84 ± 0.02 μg Cu/L (certified, 1.87 ± 0.10), 10.87 ± 0.83 μg Zn/L (11.20 ± 0.80), and 1.39 ± 0.23 μg Ni/L Ni (1.41 ± 0.12). DOC determination was carried out by an accredited lab (ALS Scandinavia AB) through IR detection according to a method based on CSN EN 1484 and CSN EN 13370. The DGT gels were eluted in 1 mL of 1 M nitric acid for 24 h and the eluate was analyzed by an ICP-MS. Three blank (unused) DGT gels were also analyzed and their average concentration was subtracted from those of the samples. The concentrations of bioavailable metals in the water, as reflected by the DGTs, were calculated following the equations provided by DGT Research Ltd. (see Supporting Information). The temperature-dependent diffusion coefficients for the calculation were selected based on the average temperature of each deployment period. Data from DGT devices found either to be covered by fouling upon retrieval, found to have been damaged during deployment, or found frozen into the sea ice, as was the case for a few devices deployed in March and November, were excluded from all calculations.

### Statistical analyses

All statistical tests were carried out in JMP 13.1.0 (significance level 5%) and the assumption of the statistical tests verified prior to their running. For all samples, values below the limit of detection were set to half the detection limit of the analysis. One-way ANOVA tests (Tukey HSD) were firstly carried out to test for significant differences in metal concentration between the different sampling points (A, B, C, or D) within the harbors, determining whether the concentrations of the sampling points could be averaged prior to any further analysis. Differences in DOC and metal concentrations (dissolved and DGT-labile) over the whole study period between the marinas and their respective reference sites were assessed through paired *t* tests. Linear regression analyses were performed between dissolved concentrations and marina occupancy, and between dissolved and DGT-labile concentrations in Bullandö Marina. Significant differences in the proportion of dissolved metals which were DGT-labile, i.e., differences in speciation, between off- and on-season at Bullandö Marina, and its reference site were evaluated with *t* tests. The on-season corresponds to the main boating season, defined here as ≥ 60% occupancy in the marina.

### Environmental guideline values and calculation of annual averages

Exceedance of EQS values, i.e., the guideline values used for the classification of water quality status in accordance with the WFD, was assessed for all investigated metals. As Ni and its compounds are on the list of so-called Priority Substances, national EQS values have been established by both Sweden and Finland. However, Cu and Zn are not Priority Substances and have only been identified as river basin–specific pollutants in Sweden. Hence, there are no Finnish EQS values for these two metals. For the environmental RA, PNEC values rather than EQS values are used as guideline values. PNEC values for marine waters have been derived at the EU level for both Cu and Zn, but given the more sensitive nature of the Baltic Sea, the competent authority in Sweden, the Swedish Chemicals Agency (SCA), uses lower PNEC values for the evaluation of Baltic Sea coatings (Table 1). Given the lack of Finnish guideline values, the concentrations at the study sites are only compared to Swedish environmental guideline values, specifically to the annual average Environmental Quality Standards (AA-EQS) and to PNEC_Baltic_ values (Table 1). Note that the concept of “added risk” is utilized for all guideline values for Zn; hence, background concentrations need to be taken into account before any comparison.

**Table 1:** Swedish annual average Environmental Quality Standards (AA-EQS) and Predicted No Effect Concentrations (PNEC) for the Baltic Sea (Swedish Agency for Marine and Water Management 2013; Swedish Chemicals Agency 2017). Also shown are the values for PNEC_marine_ derived at the EU level, for comparison (European Copper Institute 2008; European Commission 2010, 2011). All values refer to total dissolved concentrations in μg/L (0.45 μm filtered), except AA-EQS for Cu (*), which refers to the available concentration (see Eq. 1 in the “Materials and methods” section). The values for Zn are based on added risk and background values therefore need to be taken into consideration

	Cu	Zn_add_	Ni and its compounds
AA-EQS_Baltic_	0.87*	1.1	8.6
PNEC_Baltic_	1.45	2.6	-
PNEC_marine_	2.6	7.8	8.6

For the evaluation against the AA-EQS, annual average concentrations have to be calculated. For Cu, it is the available concentration, Cu_available_, which should be evaluated and calculated from Cu_diss_ with the following equation according to Swedish guidelines (Swedish Agency for Marine and Water Management 2016):

1 $$ {\mathrm{Cu}}_{\mathrm{available}}=\frac{{\mathrm{Cu}}_{\mathrm{diss}}}{{\left(\frac{\mathrm{DOC}\ }{2}\right)}^{0.6136}} $$

Cu_available_ was calculated for each time point using the concurrently sampled DOC. The annual average was then calculated from the monthly averages of the 9 (Bullandö Marina) or 3 (Porta Marina) sampled months. For Ni and Zn, the annual (dissolved) averages were calculated from monthly averages in the same way. However, following the Swedish guidelines, the background concentration, here defined as the average measured concentration at the reference site, was subtracted from each time point before generating the final average for Zn (Swedish Agency for Marine and Water Management 2016). Annual averages were therefore not calculated for the reference sites.

## Results

Statistical testing revealed no significant differences in metal concentration between the different sampling points (A, B, C, or D) within either of the marinas. The dissolved and the DGT-labile concentrations of Cu, Zn, and Ni from all sampling locations within each marina were therefore averaged together in all the graphs and in all statistical analyses.

### Dissolved concentrations

#### Dissolved metals

**Fig. 2:**
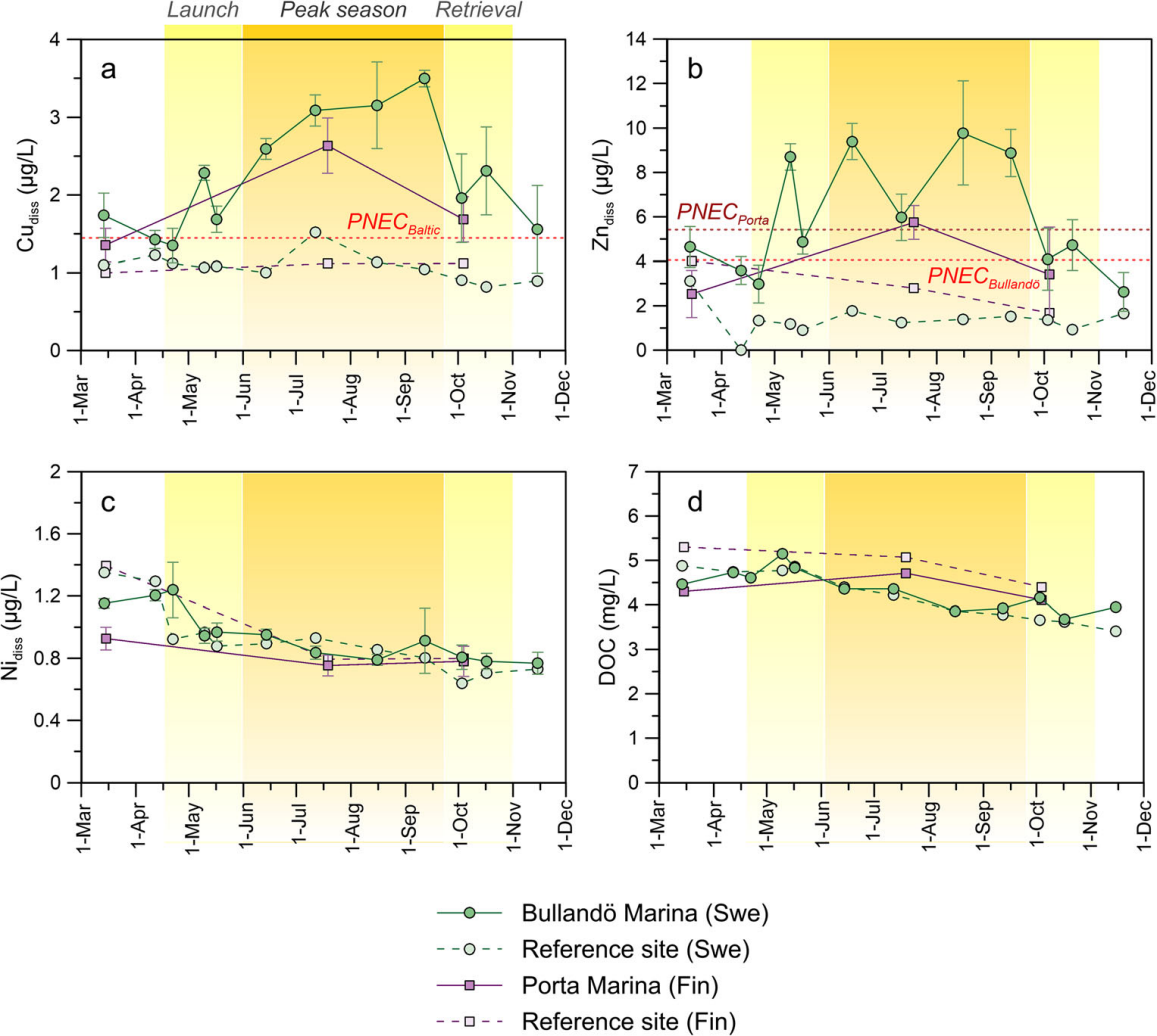
Total dissolved concentrations of Cu (a), Zn (b), and Ni (c) in the marinas (average at locations A, B, C, and D) and their respective reference sites. Error bars show the standard deviation (*n* = 4). The displayed DOC concentrations (d) were measured at one location (location A) in the marinas. The dashed red lines show the PNEC values for Cu and Zn. Note that for Zn, the background concentration (defined as the average concentration at the respective reference site) has been added to PNEC_Baltic_ for Zn_add_ (see Table 1)

The results show that concentrations of dissolved Cu (Cu_diss_, Fig. 2a) and Zn (Zn_diss_, Fig. 2b) in the marinas change over time, apparently as a function of boating activity. As Bullandö Marina was more frequently sampled than Porta Marina, the change in concentration over the whole boating season can be observed with a higher temporal resolution. The results show that Cu_diss_ starts to increase as boats were launched into the water (mid-April through May). Concentrations continue to increase until ~mid-July where they remain at a maximum (3.1–3.5 μg/L) through the peak season (defined as occupancy > 80%). As boats were retrieved from the water for winter storage in the autumn, concentrations decline to ~ 2 μg/L. Finally, at the last sampling event (Nov) when nearly all boats had been taken out of the water, concentrations are back to pre-season levels of ~ 1.5 μg/L. Zn_diss_ follows a similar pattern with lower concentrations during off-season time periods (2.6–4.6 μg/L) and elevated concentrations (6.0–9.8 μg/L) during peak season. Linear regression analysis shows that the marina occupancy, as estimated from the boat count along one of the piers, is significantly correlated to both Cu_diss_ (*p* = 0.0013, *r*^2^ = 0.649) and Zn_diss_ (*p* = 0.0033, *r*^2^ = 0.593) (fig. S1 in the Supporting Information). At the reference site, there is almost no change in Cu_diss_ and Zn_diss_, with average concentrations ± 1 SD of 1.1 ± 0.2 μg Cu/L and 1.4 ± 0.6 μg Zn/L. Paired *t* tests show that the concentrations in the marina are significantly higher than those at the reference site for both metals (*p* < 0.001). Although less frequently sampled, similar observations can be made for the Finnish sites where both Cu_diss_ and Zn_diss_ levels in Porta Marina during the peak season are comparable with those measured around the same time in Bullandö Marina and elevated compared with the Finnish reference site.

The results for Ni, a metal not associated with any type of activity related to boating, show that Ni_diss_ varies very little over the course of the study period at all sites, with average concentrations ranging from 0.8 to 1.0 μg/L. No significant difference in Ni_diss_ was found between the marinas and their corresponding reference sites for either the Swedish (*p* = 0.4440) or Finnish locations (*p* = 0.3266).

#### Dissolved organic carbon

The DOC concentrations at the marinas and their respective reference sites were comparable across the whole study period (Fig. 2d). The average concentration ± 1 SD was 4.34 ± 0.44 mg/L and 4.20 ± 0.56 mg/L for the Swedish marina and reference site, respectively. For the Finnish sites, average DOC concentrations were 4.37 ± 0.31 mg/L (marina) and 4.92 ± 0.47 mg/L (reference). Paired *t* tests revealed no significant difference in DOC concentrations between the marinas and their respective reference sites for either the Swedish (*p* = 0.1992) or Finnish (*p* = 0.1353) sites.

#### Annual averages and comparison with EQS

Annual averages as well as peak season (> 80% occupancy) averages for comparison with the Swedish AA-EQS_Baltic_ were calculated for all three metals (Table 2). For Cu, the yearly average concentrations, calculated from the monthly Cu_available_ (see Eq. 1), were found to exceed the AA-EQS_Baltic_ by factors of 1.3 (Porta Marina) and 1.7 (Bullandö Marina). During peak season, the AA-EQS_Baltic_ is exceeded roughly twice over in both marinas. Oppositely, both the annual and peak season averages for Cu at the reference sites were all below the AA-EQS_Baltic_. For Zn_add_, the concentration in Bullandö Marina was found to exceed the AA-EQS_Baltic_ approximately 3 times over the whole year and by as much as 5 times during peak season. Although the annual average at Porta Marina was just below the AA-EQS_Baltic_, the peak season concentration was 3 times that of the guideline value. Finally, for Ni, the yearly averages and peak season averages for all sites were well below the AA-EQS_Baltic_.

**Table 2:** Calculated annual and peak season (Jun–Sept) averages for Cu, Zn, and Ni at all study sites

	Cu_available_		Zn_add_		Ni	
Site	Annual average (μg/L)	Peak season (μg/L)	Annual average (μg/L)	Peak season (μg/L)	Annual average (μg/L)	Peak season (μg/L)
Bullandö Marina (Swe)	1.49	1.99	4.89	5.80	0.93	0.87
Reference (Swe)	0.69	0.76	-	-	0.92	0.87
Porta Marina (Fin)	1.16	1.56	1.07	2.91	0.78	0.75
Reference (Fin)	0.62	0.63	-	-	1.00	0.79
AA-EQS_Baltic_ (Swe)	0.87		1.1		8.6	

### Bioavailable concentrations

**Fig. 3:**
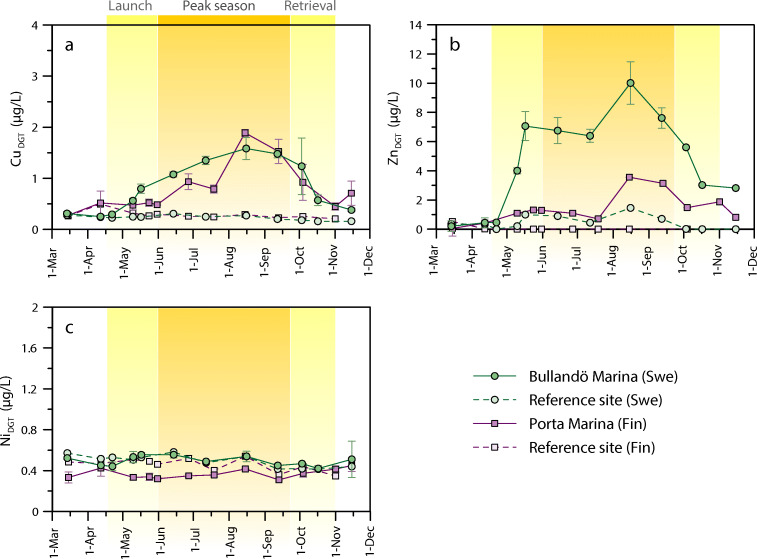
Bioavailable (DGT-labile) concentrations of Cu (a), Zn (b), and Ni (c) in the marinas (average at locations A, B, C, and D) and their respective reference sites. Error bars show the standard deviation (*n* = 4)

The time trends for the DGT-labile Cu concentrations (Cu_DGT_) closely follow those observed for Cu_diss_ at Bullandö Marina (Fig. 3a). Pre-season (March–mid-April), Cu_DGT_ is low and similar to that of the reference site at ~ 0.3 μg/L. Concentrations then increase as much as fivefold, up to 1.6 μg/L, during peak season. Similarly, the pre-season concentrations of Zn_DGT_ are low and in the same range as those measured at the reference site (~ 0.4 μg/L) (Fig. 3b). However, as the boat occupancy in the harbor increases, so does Zn_DGT_. The highest average Zn_DGT_ of 10.0 ± 1.3 μg/L is measured in mid-august, which coincides with the highest measured Cu_DGT_. Just as for the dissolved concentrations, Cu_DGT_ and Zn_DGT_ decrease as boats are retrieved from the water at the end of the boating season. Linear regression analysis shows a significant relationship for both Cu (*r*^2^ = 0.739, *p* = 0.0003) and Zn (*r*^2^ = 0.516, *p* = 0.0085) between dissolved and DGT-labile concentrations in the marina (fig. S2 in the Supporting Information). The results for Porta Marina also show an increase in Cu_DGT_ with increased boating activity, with the highest concentrations (1.9 μg/L) measured in mid- and late-August. At this time, Cu_diss_ at Porta Marina is likely to have been higher than that sampled in mid-July. Zn_DGT_ follows the same trend as Cu_DGT_, with a peak in Zn_DGT_ of 3.6 ± 0.5 μg/L in mid-August. This is nearly 3 times lower than the maximum measured at Bullandö Marina. The paired *t* tests showed significantly higher concentrations of both Cu_DGT_ (*p* = 0.0016) and Zn_DGT_ (*p* = 0.0005) in Bullandö Marina compared with its reference site. Similarly, the differences between Porta Marina and its reference site were also significant (*p* = 0.0072 for Cu and *p* = 0.0014 for Zn). For Ni_DGT_, however, the concentrations are constant across the whole study period for all sites and no significant differences could be detected between either the Swedish (*p* = 0.9905) or the Finnish (*p* = 0.3365) study locations.

**Fig. 4:**
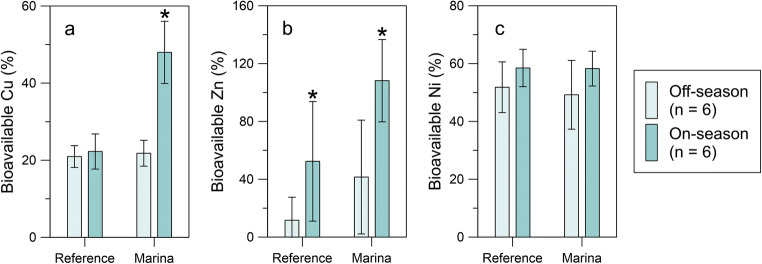
Average bioavailable proportion of dissolved Cu (a), Zn (b), and Ni (c) at Bullandö Marina (on- and off-season) and the Swedish reference site. Error bars show the standard deviation and asterisks show any significant differences between on- and off-season

At the Swedish sites, where all DGT deployments were paired with discrete water samples, the proportion of bioavailable metals in the dissolved phase was calculated (Fig. 4). At the reference site, the proportion of bioavailable Cu was on average ~ 20%, with no significant difference between on- and off-season (*p* = 0.6371). With an average of 48%, the fraction of bioavailable Cu is however significantly higher (*p* = 0.0002) in the marina during the main boating season, (≥ 60% marina occupancy, mid-May–end of September). For Zn, significant differences were established between on- and off-season for both the reference site (*p* = 0.0423) and the marina (*p* = 0.0082). The average fraction of bioavailable Zn was however found to be significantly higher at the marina compared with the reference during the on-season (*p* = 0.0409), with the majority of Zn_diss_ calculated to be in DGT-labile form (108 ± 28%). For Ni, the bioavailable fraction was ~ 50–55% for all and no significant differences were detected.

## Discussion

### Dissolved metal concentrations in marinas

The results show that both total dissolved and DGT-labile Cu and Zn concentrations were significantly elevated in the two marinas compared with their reference sites during the boating season (Figs. 2 and 3). The concentrations of Ni, on the other hand, remained more or less constant and were found to not differ between sites. There are both natural (mainly weathering) and anthropogenic (e.g., mining, metallurgical industries, and fossil fuel combustion) sources of Ni which may cause its introduction into waters from air and/or adjacent land (European Commission 2008). Hence, any increase in Cu and Zn concentration cannot be explained here by differences between marina and reference sites in, for instance, the amount of runoff from land. The results clearly suggest that the elevated concentrations in the marina waters are related to boating activity, with antifouling paints acting as the main sources of Cu and Zn. Sacrificial zinc anodes may also be an additional source of Zn to the marina waters (Bird et al. 1996; Rees et al. 2017), as will be discussed further on.

The water exchange at Porta Marina is likely higher than that at Bullandö Marina as it is less enclosed (Fig. 1). Additionally, with only half as many berths, one would expect lower concentrations in the Finnish marina. However, both Cu_diss_ (Fig. 2a) and Cu_DGT_ (Fig. 3a) were comparable in the two marinas during peak boating season. This could be explained by the fact that paints with higher leaching rates of Cu are allowed to be marketed in Finland. As for Zn, DGT-labile concentrations are indeed lower at Porta Marina than Bullandö Marina (Fig. 3b), which could be the result of differences in water exchange and number of boats. Nevertheless, Zn_diss_ at Bullandö Marina is very elevated, with concentrations of as much as ~ 10 μg/L. This is considerably higher than Cu_diss_ (maximum of 3.5 μg/L) and cannot be explained by differences in background levels between the two metals. Due to the stricter Swedish RA, the authorized paints to be used in the Swedish part of the Baltic Sea contain lower amounts of cuprous oxide (up to 8.5 wt% at the time of the study) than those authorized for other EU countries, e.g., Finland (up to 40 wt%). However, the Swedish Baltic Sea coatings often tend to contain higher amounts of zinc oxide instead. The in situ release rates of Cu and Zn for three antifouling paints authorized on the Swedish East coast, i.e., the Baltic Sea, were measured in Bullandö Marina in 2015 (Lagerström et al. 2018). The measured release rates (in μg cm^−2^ day^−1^) showed that on average twice as much Zn was released relative to Cu.

### Comparison with other marinas and EQS values

The measured Cu_diss_ and Zn_diss_ at the two marinas during peak season can be compared with measurements from other marinas located in the same region and sampled at similar times of the year (Table 3). The lowest measured concentrations are typically found in the marinas with less intense boating activity, i.e., the natural and guest harbors. However, data from the much enclosed and lagoon-shaped natural harbor of Säck (59.3904° N, 8.7983° E) shows that concentrations as high as 5.75 μg Cu/L and 11.8 μg Zn/L can occasionally be measured during peak season. The other sampled marinas hold concentrations of 2.0–3.9 μg Cu/L and 7.1–10.6 μg Zn/L. These ranges are similar to those of the two marinas in this study, with measured peak season ranges of 2.6–3.5 μg Cu/L and 5.0–9.8 μg Zn/L. In Baltic Sea field experiments, similar concentrations of Cu and Zn were strongly correlated to increased mortality and reduced reproduction in exposed organisms (Bighiu et al. 2017a, 2017b).

**Table 3:** Concentrations of dissolved Cu and Zn at marinas located in the Stockholm and Turku archipelagos

Location	Sampling time and depth	Number of moorings	Cu_diss_ (μg/L)	Zn_diss_ (μg/L)	Reference
Natural harbor (Swe) *Säck*	Jun–Sept, 2004 (0.5-m depth)	-	0.73–5.75	0.65–11.8	(Kylin and Haglund 2010)
Natural harbor (Swe) *Säck*	August, 2006 (1-m depth)	-	0.90	-	(Ndungu 2012)
Guest harbor (Swe)	Summer, 2014 and 2015 (1-m depth)	390	1.4–1.8	1.8–3.3	(Bighiu et al. 2017a)
Marina (Fin) *Uittamo Marina*	Summer, 2005 (surface)	250	2.0–3.89	-	(Brooks 2006)
Marina (Swe) *Marina 1*	Summer, 2014 and 2015 (1-m depth)	1400	3.5–3.7	7.1–10.6	(Bighiu et al. 2017a)
Marina (Swe) *Marina 2*	Summer 2015 (1-m depth)	1400	2.7	8.1	(Bighiu et al. 2017a)

The comparison of the calculated annual average concentrations in the marinas to the Swedish AA-EQS_Baltic_ typically showed exceedance for both Cu and Zn, with highest exceedance during peak season (Table 2). Hence, the marina waters fail to achieve “Good Status” with respect to these specific pollutants. The comparable concentrations of Cu_diss_ and Zn_diss_ measured in several other harbors (Table 3) suggest that this will likely be the case for many other Baltic Sea marinas. There are roughly 450 coastal water bodies in Sweden where the use of biocidal paints on leisure boats is permitted (between Örskär in the Baltic Sea and the Norwegian border on the West coast). GIS analysis with map layers generated for an inventory of recreational marinas and jetties along the Swedish coast in 2008 (Swedish EPA 2010) shows that 72% of these coastal water bodies hold at least one marina. The number of marinas varies from 1 to 23, with an average of 4.3 marinas per water body. Additionally, there are on average 151 jetties (with a maximum of 1527 jetties) per water body, outside of those located within marinas. Given this intense coastal exploitation, the use of biocidal antifouling paints has the potential to impact the status of many water bodies, especially as the numbers of marinas and jetties have likely increased since 2008. According to the latest classification (2018-09-09), only 6 (1.3%) of these coastal water bodies meet the criteria for good ecological status (VISS 2018).

### Effect on metal speciation

In Bullandö Marina, the concentrations of bioavailable Cu, Zn, and Ni follow the same time trends as the dissolved concentrations (Fig. 3). However, the proportion of bioavailable to total dissolved concentrations is shown to change significantly with increased boating activity for Cu and Zn (Fig. 4). This shift in speciation is not accompanied by any change in DOC concentration (Fig. 1d). In open ocean and coastal surface waters, Ni and Zn are reported to form weaker complexes with organic ligands than Cu (van den Berg et al. 1987; Vraspir and Butler 2009; Whitby and van den Berg 2015; Boiteau et al. 2016). The Irving-Williams order describes the relative stability of metal complexes with organic ligands which, in descending order for the studied metals, is Cu > Zn ≈ Ni (Irving and Williams 1953). In accordance with this order, the proportion of bioavailable metals at the marina during the off-season and at the reference site show that the highest affinity for organic ligands is indeed observed for dissolved Cu (~ 80% is non-bioavailable), followed by Zn (~ 60%) and Ni (~ 50%). The fact that no speciation change is detected for Ni between on- and off-season suggests that the effect seen for Cu cannot be explained by lower availability of ligands, given that Cu has a higher affinity for organic ligands than Zn and Ni. The increased proportion of bioavailable Cu and Zn during the boating season may instead be explained by reaction kinetics. The results from this study could suggest that the metal release from antifouling paints may occur at a rate higher than that of the complexation reactions with ligands.

Previous studies have been carried out in marinas to investigate seasonal variations in Cu speciation. Jones and Bolam (2007) studied concentrations of labile (free and inorganically complexed, determined through anodic stripping voltammetry) and dissolved Cu in three estuarine UK marinas (16.5–34.2 PSU) in 2001–2002. Although seasonal changes in dissolved Cu were comparable with those found here (rising concentrations from winter to late summer and decreasing during autumn/winter), no change in speciation was detected. This was attributed to the presence of complexing agents and suspended particles able to bind labile Cu and prevent its build-up in the water. The change in Cu speciation was similarly studied over an annual cycle in a Finnish marina in 2005 without any observed change in speciation (Brooks 2006). However, samples were only collected once per season in both studies, which may not have been frequent enough to detect differences between on- and off-season. If the same limited number of time points had been used in the current study, it is not certain that the change in speciation would have been detected. The added risk associated with the increased proportion of bioavailable metals due to the emissions from antifouling paints needs to be investigated further. The current RA procedure of antifouling paints is only concerned with the concentrations of total dissolved Cu and Zn but should a change in speciation be confirmed in more marinas, there might be a need to revise this practice.

### Is the risk assessment of antifouling paints adequate in protecting Baltic Sea waters?

#### Finland

MAMPEC modeling is only utilized by one of the countries in this study: Sweden, where the Swedish Chemicals Agency (SCA) is the competent authority. The Finnish authority Tukes does not evaluate PECs derived in MAMPEC but has instead a limit value for the leaching rate of Cu of 15 μg cm^−2^ day^−1^ over any 14-day period (Tukes 2018). Any release of Zn is currently not taken into consideration. In comparison, the Swedish RA for Baltic Sea waters is such that the steady-state release rate of Cu cannot exceed ~ 1 μg cm^−2^ day^−1^ in order to obtain PEC/PNEC_Baltic_ < 1. Paints with higher leaching rates of Cu are therefore allowed to be marketed in Finland compared to Sweden, even though both countries border the same sea, with similar sensitivity and fouling pressure. The concentration measured at the one sampling time during peak season (mid-July) in the Finnish marina shows that both Cu_diss_ and Zn_diss_ exceed PNEC_Baltic_ (Fig. 2a, b). The DGT results additionally suggest that concentrations would likely have been even higher if measured in mid-August. Given this and the fact that Porta Marina is not a very enclosed marina, i.e., should have a fairly good water exchange rate, suggests that the current Finnish leaching requirement is not adequately protecting the marine environment.

#### Sweden

**Fig. 5:**
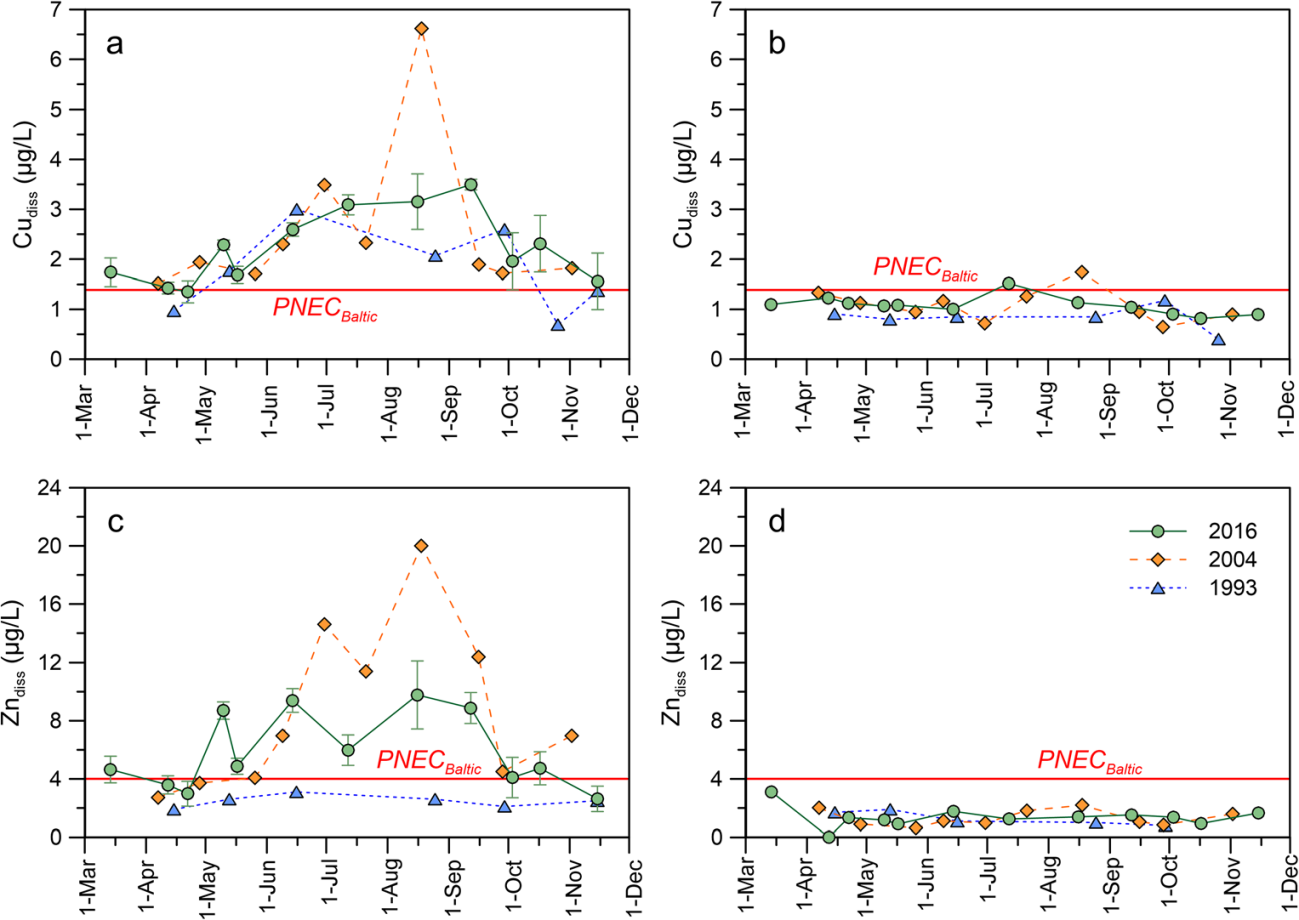
Dissolved concentrations of Cu (a) and Zn (c) measured in Bullandö Marina in this study (2016) and at station 10 (see Fig. 1b) during two previous studies in 1993 [58] and 2004 [13]. Concentrations of Cu (b) and Zn (d) at nearby reference sites were also measured during each study. The red lines show the Swedish PNEC values for the Baltic Sea

Bullandö Marina in Sweden was sampled more frequently, allowing for a more comprehensive evaluation. Cu_diss_ and Zn_diss_ have also been measured in this marina and various nearby reference sites in previous years, giving further insight into how legislation and RA procedures have affected marina concentrations over the years (Fig. 5). The measurements show that background concentrations have not changed between 1993 and 2016, with yearly averages of 0.8–1.1 μg Cu/L and 1.3–1.4 μg Zn/L (Fig. 5b, d). Despite changes in legislation and refinement of the RA procedure of antifouling paints over the past 25 years, concentrations of Cu_diss_ in the marina have not markedly changed (note that the number of boats moored in the marina was the same for all years). As of 2001, biocidal antifouling paints for use on recreational boats in the Baltic Sea were no longer allowed to be sold in Sweden as the environmental risks were deemed to outweigh the benefits (Swedish Chemicals Agency 1998). However, Cu_diss_ measured in 2004 is not distinctly lower compared to 1993, suggesting perhaps a continued (illegal) use of copper-containing antifouling paints. A refined environmental RA procedure utilizing the MAMPEC model was later introduced, resulting in the authorization of copper-based antifouling paints in the Baltic Sea again as of 2011. Bullandö Marina is the Swedish MAMPEC marina used to model PECs for the RA of antifouling paints intended for use in the Baltic Sea (Ambrosson 2008). The exceedance of PNEC_Baltic_ observed for Cu_diss_ in 2016 is therefore surprising.

There are some clear differences between years when it comes to Zn_diss_. The higher concentrations observed in 2004 compared to 1993 could be explained by the introduction of so-called biocide-free paints containing ZnO and with high leaching rates of Zn on the market during the ban of biocidal paints (Ytreberg et al. 2010). The zinc-based paints, which did not require any authorization as they were deemed to deter fouling through physical and not chemical means, were later found to be highly toxic (Karlsson et al. 2010) and removed from the market by 2012 (Swedish Chemicals Agency 2018). Consequently, this could explain the comparatively lower Zn_diss_ measured in 2016. Concentrations in 2016 during peak season are however still 2–3 times higher than those measured in 1993. This could be due to a higher content of ZnO in the copper-based antifouling paints authorized at the time of the study, as previously mentioned (see the “Dissolved metal concentrations in marinas” section). Just as for Cu_diss_, Zn_diss_ also exceeded PNEC_Baltic_ in 2016.

There could be several possible explanations for the > 2-fold exceedance of PNEC_Baltic_ in Bullandö Marina for the two metals: (1) illegal use of paints with higher leaching rates of Cu and Zn than allowed, (2) existence of additional sources of Cu and Zn to the marina waters, and/or (3) the RA is not refined enough, leading to the authorization of antifouling paints with leaching rates that cause exceedance of PNEC_Baltic_. In a survey from 2015, only 4% of boat owners moored within this section of the Swedish East coast stated that they were using a paint not authorized for the Baltic Sea, whereas 88% stated they were using a lawful paint and 8% did not know (The Swedish Transport Agency 2015). Thus, any illegal use of paint (1) is likely not significant enough to explain the observed concentrations. Whereas no additional sources of Cu to the marina waters are known (2), sacrificial zinc anodes could contribute in part to the observed Zn_diss_ in the marina. In a recent study in the Hamble estuary in the UK, anodes were hypothesized to be the main source of Zn_diss_ to the river (Rees et al. 2017). In Bullandö Marina, however, the low Zn_diss_ measured in 1993 (Fig. 5c) is only on average 1.6 μg/L higher than the reference. Even if one assumes that all of this added Zn was solely attributed to the leaching from sacrificial anodes, it is not enough to explain the exceedance of the PNEC_Baltic_ by roughly 4.5 μg/L during the 2016 peak season. Furthermore, in brackish and low salinity waters, aluminum anodes may be more commonly used as they are both longer lasting and provide better corrosion protection (Wigg and Fleury 2012; Rees et al. 2017). To evaluate the last possible explanation, i.e., the performance of the RA (3), one needs to investigate whether the MAMPEC model yields representative PECs.

#### MAMPEC evaluation

**Fig. 6:**
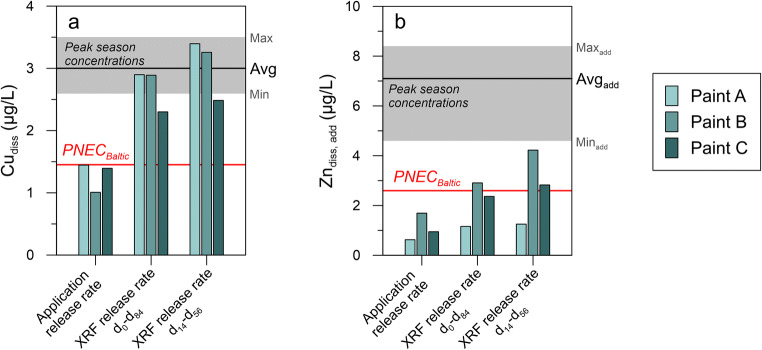
Predicted environmental concentrations (PECs) in Bullandö Marina derived in MAMPEC for Cu (a) and Zn (b) for three Swedish Baltic Sea paints. Shown here are the PECs from the product application reports. PECs derived from XRF release rates, as measured in Bullandö Marina in 2015, are also shown [42]. Both the average release rates measured between exposure days 0–84 and days 14–56 were used and PECs were derived using the same MAMPEC parameters, as specified in Lagerström et al. (2018). The shaded gray area shows the concentration range measured during peak boating season in 2016 (marina occupancy > 80%), with the black line showing the average. The red line shows PNEC_Baltic_. Note that the concentrations of Zn_diss,add_ refer to the anthropogenically added concentrations; hence, the average concentration measured at the reference site (1.4 μg/L) has been subtracted from the peak season concentrations

The Swedish MAMPEC marina scenario for antifouling paints intended for use in the Baltic Sea is tailored after Bullandö Marina. The field measurements in this marina can thus be used to evaluate whether the PECs derived in MAMPEC are indeed realistic. There are several parameters that can affect the accuracy of the generated PECs. For one, the only input parameter which will vary between paints is the release rate and it has long been questioned whether the currently standardized methods yield representative rates (IMO 2009). To investigate this potential source of error, PECs were derived in MAMPEC using release rates derived with different methods for 3 out of the 7 antifouling paints authorized for use in the Baltic Sea at the time of the study (Fig. 6). For both Cu and Zn, the release rates used in the application to the SCA for product approval, i.e., using standardized methods, yield PECs ≤ PNEC_Baltic_. This is not surprising, as the paints would likely not have been authorized otherwise. The modeled PECs are however well below the observed concentrations in the marina. Oppositely, if in situ release rates measured in Bullandö Marina and determined through X-ray fluorescence (XRF) analysis in a study from 2015 (Lagerström et al. 2018) are used instead, the PECs for Cu are much more on par with observed concentrations. Another parameter that could also contribute to an underestimation of the PECs for Cu, although to a lesser degree, is the background concentration. The Swedish PEC calculation currently assumes a background concentration of 0.69 μg/L, which is slightly low given the observed range of 0.8–1.1 μg/L at the nearby reference sites between 1993 and 2016.

A similar comparison of release rate inputs into the MAMPEC model was carried out for Zn. Regardless of the release rate method, none of the calculated PECs is sufficiently high to account for the measured concentrations of Zn (Fig. 6b). One of the compound input parameters in MAMPEC, the particulate/dissolved partitioning coefficient *K*_D_, could be the main source of error here. For Cu, a *K*_D_ based on compiled measurements in estuarine and brackish waters and the North Sea is used for the Swedish MAMPEC scenarios, but this is not the case for Zn. For the three paints, the *K*_D_ used to derive the PEC for the product authorization application was either 126 (paint B) or 110 m^3^/kg (A and C). The origin of the former is unknown, but the latter comes from the EU’s risk assessment report for zinc where it is reported as the median *K*_D_ measured in Dutch river waters between 1983 and 1986 (European Commission 2010). If *K*_D_ values for Zn are instead compiled from the same studies as those used to determine that for Cu in estuarine and brackish waters (European Copper Institute 2008), the median is calculated to 44 m^3^/kg (Table S1, Supporting Information). With this *K*_D_, the calculated PECs for Zn increase ~ 3-fold, yielding averages for the three paints of 3.3 ± 1.9 (using the application release rates), 6.9 ± 2.9 (XRF release rate, days 0–84), and 8.9 ± 4.8 μg/L (XRF release rate, days 14–56). Again, the in situ release rates yield the most realistic PECs.

#### Other marine waters in the EU

Data on dissolved copper concentrations in recreational marinas in other marine waters in the EU are scarce, as most studies tend to focus on the sediment compartment and not the dissolved phase. In fact, only two peer-reviewed articles published in the last 15 years were found during the literature search. In the UK, Cu_diss_ in three estuarine marinas was found to occasionally exceed PNEC_marine_ (2.6 μg/L), with measurements ranging from 1.6 to 4.4 μg/L in the spring/summer of 2001 (Jones and Bolam 2007). Similarly, a study of copper concentrations in German marinas (11 brackish and 5 saltwater) sampled during the summer season of 2013 revealed median and maximum Cu_diss_ concentrations of 5 μg/L and 20 μg/L, respectively (Daehne et al. 2017). A majority of the German marinas thus exceeded PNEC_marine_. Hence, the need for a refined RA of antifouling paints is likely not restricted to the Baltic Sea.

## Conclusions

The seasonal variations of Cu and Zn (dissolved and bioavailable) at the two Baltic Sea marinas show clearly how the use of antifouling paints increases the metal concentrations in the water during the boating season, leading to exceedance of both EQS and PNEC values. The significant changes in speciation caused by antifouling paints, shown for the first time in this study, also indicate that the environmental risks associated with these products could be underestimated when only total dissolved concentrations are taken into consideration. More speciation studies need to be carried out in marinas in order to confirm the findings of this study.

The failure in yielding representative PECs for Bullandö Marina in MAMPEC reveals some key issues with the current procedure. Most importantly, the comparison with measured concentrations shows that representative release rates must be used in order to yield realistic PECs. Additionally, the *K*_D_ used for Zn for the Baltic Sea scenario should be revised. The evaluation of the PEC derivation was only made possible by the fact that Bullandö Marina is the marina after which the Swedish East coast marina scenario is tailored. This highlights the importance of MAMPEC scenarios based on real marinas, enabling the validation of model results and thus ensuring the relevancy of the environmental RA. Although the scope of this study is limited to the Baltic Sea, dissolved Cu concentrations reported from recreational marinas in other marine waters suggest the need for an overall refinement of the environmental RA within the EU.

## Electronic supplementary material

ESM 1 (PDF 268 kb)
