# Supplementary material for: Flawed risk assessment of antifouling paints leads to exceedance of guideline values in Baltic Sea marinas
Source: Environ Sci Pollut Res Int. 2020 May 11;27(22):27674–87. doi: 10.1007/s11356-020-08973-0 (PMC7334261; doi:10.1007/s11356-020-08973-0)
Supplement: Supplementary file 1 — (PDF 268 kb) [file 11356_2020_8973_MOESM1_ESM.pdf]

Supporting Information

Flawed risk assessment of antifouling paints leads to exceedance of guideline values in Baltic Sea marinas

Maria Lagerström[a], e-mail: maria.lagerstrom@aces.su.se

João Ferreira[a], e-mail: joao.l.g.ferreira@gmail.com

Erik Ytreberg[b], e-mail: erik.ytreberg@chalmers.se

Ann-Kristin Eriksson Wiklund[a], e-mail: ann-kristin.eriksson-wiklund@aces.su.se

[a] *Department of Environmental Science and Analytical Chemistry (ACES), Stockholm University, SE-106 91 Stockholm, Sweden.*

[b] *Department of Mechanics and Maritime Sciences, Chalmers University of Technology, SE 412 96 Gothenburg, Sweden*

*Corresponding author: Maria Lagerström, e-mail: maria.lagerstrom@aces.su.se

Pages: 3

Figures: 2

Tables: 1

2. Materials and Methods

2.3. Chemical analyses

The DGT-labile concentrations (C_{DGT}) in the water were derived from the eluate concentrations using the two equations below provided by DGT Research LTD (https://www.dgtresearch.com/guides-to-using-dgt/):

$$M = C_e \times \frac{V_{HNO_3} + V_{gel}}{f_e}$$

where M is the mass of metal accumulated in the resin gel layer (g), C_e is the concentration of metals in the eluate (g L^{-1}), V_{HNO3} is the volume of 1M HNO_3 added to the resin gel (0.001 L), V_{gel} is the volume of the resin gel (0.0015 L), and f_e is the elution factor for the metal (equal to 0.8).

$$C_{DGT} = \frac{M\Delta g}{DtA}$$

where Δg is the thickness of the diffusive gel (0.078 cm) plus the thickness of the filter membrane (0.014 cm), D is the diffusion coefficient of metal in the gel (in cm^2 sec^{-1}, https://www.dgtresearch.com/diffusion-coefficients/), t is deployment time (sec) and A is the exposure area (3.14 cm^2).

3. Results

3.1. Dissolved concentrations

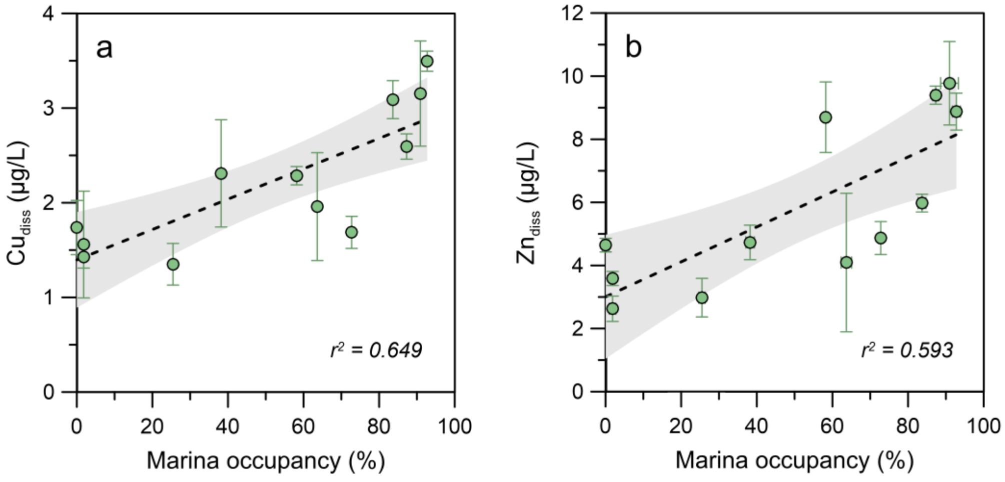

Figure S1. Plots of dissolved Cu (a) and Zn (b) against the % occupancy at Bullandö Marina. Error bars show the standard deviation (n=4). The dashed line shows the linear regression and the grey shaded area shows the 95% confidence interval.

3.2. Bioavailable concentrations

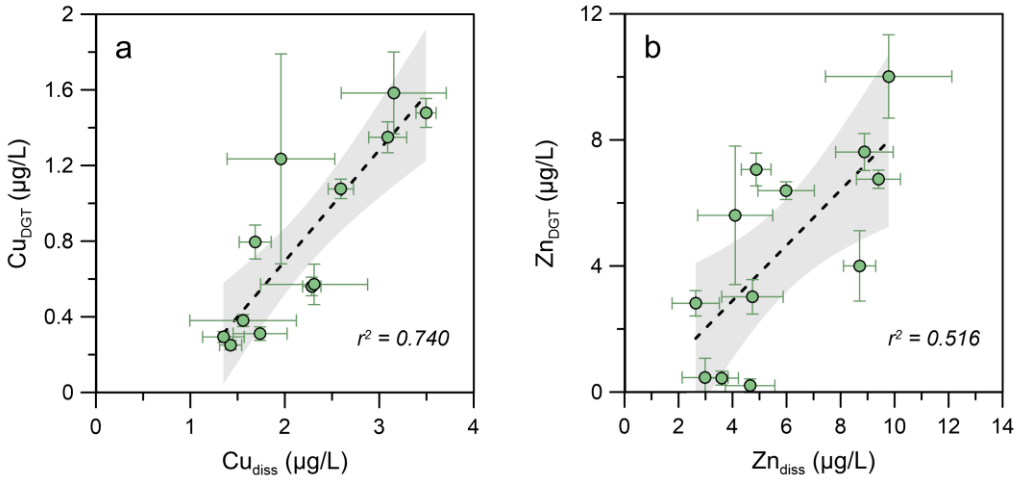

Figure S2. Plots of dissolved against bioavailable concentrations of Cu (a) and Zn (b) at Bullandö Marina. Error bars show the standard deviation (n=4). The dashed line shows the linear regression and the grey shaded area shows the 95% confidence interval.

4. Discussion

4.4. Is the risk assessment of antifouling paints adequately protecting marine waters?

Table S1. K_D-values used for the derivation of a typical estuarine/brackish K_D for Zn, based on the same studies used to derive the estuarine/brackish K_D for Cu in [1].

Area/Estuary	Log Zn K_D (mean)	Reference
**Forth Estuary**	4.88	[2]
**Tay Estuary**	5.16	[2]
**Rhine**	4.58	[3] (in: [4])
**Waal**	4.64	[3] (in: [2])
**Maas**	4.64	[3] (in: [2])
**Weser**	4.77	[4] (in: [2])
**Seine**	4.6	[5] (in: [2])
**Mersey**	4.5	[6] (in: [2])
**Humber**	4.4	[6] (in: [2])
**Baltic Sea**	*	[7]
**Scheldt**	4.34	[8–10]
**Rhone**	**	[11]
**Lena**	5.7	[12]
**North sea estuaries**	4.5	[13] (in: [8])
**Conwy estuary**	4.65	[14]
**San Francisco Bay**	6.60	[15]
**Six estuaries in Texas, USA**	5.05	[16]
**Median:**	4.64	

*K_D for Zn not determined

** Reference could not be found

References

[1] European Copper Institute, Voluntary Risk Assessment Report (VRAR) for copper, copper(II) sulfate pentahydrate, copper(I) oxide, copper(II) oxide, dicopper chloride trihydroxide., 2008.
[2] R.E. Owens, P.W. Balls, N.B. Price, Physicochemical processes and their effects on the composition of suspended particulate material in estuaries: Implications for monitoring and modelling, Mar. Pollut. Bull. 34 (1997) 51–60. doi:10.1016/S0025-326X(96)00054-9.
[3] J. Golimowski, A.G.A. Merks, P. Valenta, Trends in heavy metal levels in the dissolved and particulate phase in the Dutch rhine-meuse (MAAS) delta, Sci. Total Environ. 92 (1990) 113–127. doi:10.1016/0048-9697(90)90324-N.
[4] A. Turner, G.E. Millward, B. Schuchardt, M. Schirmer, A. Prange, Trace metal distribution coefficients in the Weser Estuary (Germany), Cont. Shelf Res. 12 (1992) 1277–1292. doi:10.1016/0278-4343(92)90064-Q.
[5] J.-F. Chiffoleau, D. Cossa, D. Auger, I. Truquet, Trace metal distribution, partition and fluxes in the Seine estuary (France) in low discharge regime, Mar. Chem. 47 (1994) 145–158. doi:10.1016/0304-4203(94)90105-8.
[6] S.D.W. Comber, A.M. Gunn, C. Whalley, Comparison of the partitioning of trace metals in the Humber and Mersey estuaries, Mar. Pollut. Bull. 30 (1995) 851–860. doi:10.1016/0025-326X(95)00092-2.
[7] C. Pohl, U. Hennings, The effect of redox processes on the partitioning of Cd, Pb, Cu, and Mn between dissolved and particulate phases in the Baltic Sea, Mar. Chem. 65 (1999) 41–53. doi:10.1016/S0304-4203(99)00009-2.
[8] R.F. Nolting, W. Helder, H.J.W. de Baar, L.J.A. Gerringa, Contrasting behaviour of trace metals in the Scheldt estuary in 1978 compared to recent years, J. Sea Res. 42 (1999) 275–290. doi:10.1016/S1385-1101(99)00036-2.
[9] H. Paucot, R. Wollast, Transport and transformation of trace metals in the scheldt estuary, Mar. Chem. 58 (1997) 229–244. doi:10.1016/S0304-4203(97)00037-6.
[10] F. Monteny, M. Elskens, W. Baeyens, The behaviour of copper and zinc in the Scheldt estuary, Netherland J. Aquat. Ecol. 27 (1993) 279–286. doi:10.1007/BF02334791.
[11] P. Regnier, M. Hoenig, L. Chou, R. Wollast, Trace metals in the suspended matter collected in the mixing zone of the Rhone estuary, Water Pollut Res Rep. 20 (1990) 385–396.
[12] J.M. Martin, D.M. Guan, F. Elbaz-Poulichet, A.J. Thomas, V.V. Gordeev, Preliminary assessment of the distributions of some trace elements (As, Cd, Cu, Fe, Ni, Pb and Zn) in a pristine aquatic environment: The Lena River estuary (Russia), Mar. Chem. 43 (1993) 185–199. doi:10.1016/0304-4203(93)90224-C.
[13] P.W. Balls, The partition of trace metals between dissolved and particulate phases in european coastal waters: A compilation of field data and comparison with laboratory studies, Neth. J. Sea Res. 23 (1989) 7–14. doi:10.1016/0077-7579(89)90037-9.
[14] J.L. Zhou, Y.P. Liu, P.W. Abrahams, Trace metal behaviour in the Conwy estuary, North Wales, Chemosphere. 51 (2003) 429–440. doi:10.1016/S0045-6535(02)00853-6.
[15] S.A. Sañudo-Wilhelmy, I. Rivera-Duarte, A. Russell Flegal, Distribution of colloidal trace metals in the San Francisco Bay estuary, Geochim. Cosmochim. Acta. 60 (1996) 4933–4944. doi:10.1016/S0016-7037(96)00284-0.
[16] G. Benoit, S.D. Oktay-Marshall, A. Cantu, E.M. Hood, C.H. Coleman, M.O. Corapcioglu, P.H. Santschi, Partitioning of Cu, Pb, Ag, Zn, Fe, Al, and Mn between filter-retained particles, colloids, and solution in six Texas estuaries, Mar. Chem. 45 (1994) 307–336. doi:10.1016/0304-4203(94)90076-0.
